# The role of cytoplasmic p57 in invasion of hepatocellular carcinoma

**DOI:** 10.1186/s12876-015-0319-x

**Published:** 2015-08-15

**Authors:** Hui Guo, Yi Li, Tao Tian, Lili Han, Zhiping Ruan, Xuan Liang, Wenjuan Wang, Kejun Nan

**Affiliations:** Department of Oncology, The First Affiliated Hospital, College of Medicine of Xi’an Jiaotong University, No. 277 Yanta West Road, Yanta District, Xi’an, Shaanxi Province 710061 P.R. China

**Keywords:** Cytoplasmic p57, Invasion, Hepatocellular carcinoma

## Abstract

**Background:**

Our previous research suggested that p57 downregulation could accelerate the growth and invasion of hepatocellular carcinoma *in vitro* and *in vivo*.

**Aim:**

To evaluate the role of cytoplasmic p57 and its regulatory mechanism during hepatocellular carcinoma invasion.

**Methods:**

We examined the subcellular localization of p57 by immunohistochemistry in 45 pairs of cancerous tissues and adjacent non-cancerous tissues. Moreover, we generated stable *p57* knockdown hepatoma cell lines to investigate the mechanism of cytoplasmic p57-mediated regulation of invasion by immunoprecipitation, confocal immunofluorescence microscopy and western blot of nuclear and cytoplasmic extracts.

**Results:**

Our results showed that cytoplasmic expression of p57 was reduced in specimens from patients with capsular invasion and metastasis (*P* < 0.05). Moreover, the level of p-cofilin was decreased in the group lacking cytoplasmic p57 expression (*P* < 0.05). Co-expression of p57 and p-cofilin was reduced in specimens from patients with tumors at later stages (III + IV), tumors showing capsular invasion and metastatic tumors. We further observed that p57 downregulation decreased the assembly of p57 and LIM domain kinase 1 and its kinase activity, subsequently reducing the level of p-cofilin in the cytoplasm.

**Conclusions:**

Cytoplasmic p57 might be a key regulator in hepatocellular carcinoma invasion via the LIM domain kinase 1/p-cofilin pathway.

**Electronic supplementary material:**

The online version of this article (doi:10.1186/s12876-015-0319-x) contains supplementary material, which is available to authorized users.

## Background

p57 is a cyclin-dependent kinase inhibitor (CKI) that belongs to the Cip/Kip family, which includes p21, p27 and p57. The human *p57* gene is located on chromosome 11p15.5, a region implicated in Beckwith-Wiedemann syndrome, a familial cancer syndrome [1]. Low expression of p57 protein has been observed in human tumors, including carcinomas of the prostate, bladder, gastrointestinal tract, pancreas and breast, which suggests that p57 is an important tumor suppressor [2–4]. An increasing number of studies have shown that p57 is a multifunctional protein that is also involved in the regulation of transcription, apoptosis, differentiation and motility [5, 6].

Whereas the function of Cip/Kip proteins as inhibitors of the cell cycle is well characterized in the nucleus, these proteins also seem to regulate cytoskeletal function in the cytoplasm. The Cip/Kip proteins have been reported to modulate the RhoA/ROCK/LIMK/cofilin signaling pathways involved in tumor invasion and metastasis. Surprisingly, p21 and p27 have been shown to interact with Ras homolog gene family member A (RhoA) and rho-associated, coiled-coil-containing protein kinase 1 (ROCK1) in the cytoplasm and to accelerate cancer cell invasion and metastasis, suggesting a role for these proteins that opposes the typical role of tumor suppressors in the nucleus [7]. Although several studies have reported that p57 can interact with LIM domain kinase 1 (LIMK1) to affect actin cytoskeleton dynamics, the role of cytoplasmic p57 in the regulation of tumor cell invasion is unclear [8].

Hepatocellular carcinoma (HCC) is the sixth most prevalent cancer in the world and the third leading cause of cancer-related mortality [9]. The main reason for the poor prognosis observed in HCC patients is the highly proliferative and metastatic activities of HCC cells, which are the result of the deregulation of multiple signaling pathways [10]. Our previous research suggests that the downregulation of p57 accelerates the growth and invasion of HCC, indicating that p57 is an important tumor suppressor in HCC development [11]. Moreover, cytoplasmic expression of p57 was observed in HCC tissue and HCC cell lines. We are thus interested in further investigating the role and mechanism of cytoplasmic p57 in the invasion of HCC.

## Methods

### Patients and samples

A total of 45 paired HCC specimens and adjacent non-cancerous specimens were obtained from surgical resections performed at the First Affiliated Hospital, College of Medicine, Xi’an Jiaotong University. The study participants included 36 males and 9 females with a mean age of 48.33 ± 10.55 years (range, 29–77 years). The other clinicopathologic data are presented in Table 1. The pathological type of each specimen was confirmed by independent pathologists. None of the patients received radiotherapy or chemotherapy prior to surgery. The TNM stage was determined using the 2002 Union International Centre Cancer (UICC) criteria. Tumor cellular differentiation was identified by Edmondson’s classification. The study was approved by the Conduct of Human Ethics Committee of the First Affiliated Hospital, College of Medicine, Xi’an Jiaotong University. Informed consent was obtained from each patient.

**Table 1:** Relationship between the subcellular distribution of p57 protein and clinicopathologic factors in cancerous tissues of hepatocellular carcinoma patients

Variable	n	Nuclear p57 ($\overline{x}$ ± s)	*P*	Cytoplasmic p57 ($\overline{x}$ ± s)	*P*
Tumor size			0.016^*^		0.199
<50 mm	18	4.82 ± 3.10		4.32 ± 2.92	
≥50 mm	27	2.88 ± 2.65		3.18 ± 3.33	
Histological grade			0.283		0.503
I + II	36	3.88 ± 3.08		3.79 ± 3.17	
III	9	2.83 ± 2.48		3.08 ± 3.37	
TNM stage			0.150		0.098
I + II	12	4.60 ± 3.07		4.80 ± 3.57	
III + IV	33	3.30 ± 2.89		3.20 ± 2.97	
Capsular invasion and extrahepatic metastasis			0.05		0.013
Positive	24	2.97 ± 2.86		2.71 ± 3.10	
Negative	21	4.54 ± 2.93		4.83 ± 2.96	
AFP			0.131		0.670
<400 μg · L^−1^	21	4.32 ± 2.84		3.84 ± 3.13	
≥400 μg · L^−1^	24	3.10 ± 3.01		3.47 ± 3.29	

*n* Number of patients, *AFP* alpha-fetoprotein, *TNM* tumor-node-metastasis

### Histopathology and immunohistochemistry

Paraffin-embedded sections were deparaffinized and rehydrated. For histopathology, the sections were stained with hematoxylin/eosin (HE). For immunohistochemistry, antigens were retrieved in citrate buffer, and the sections were blocked with 3 % hydrogen peroxide and incubated with a primary antibody against p57 (1:100, sc-56341, Santa Cruz Biotechnology, Santa Cruz, CA, USA) and anti-p-cofilin (Ser3) antibody (1:100, 11139, Signalway Antibody, Pearland, TX, USA) overnight at 4 °C. Control sections were incubated with an isotype-matched control antibody. Next, the sections were incubated with biotin-conjugated secondary antibodies for 30 min and streptavidin-peroxidase for 30 min. Immunoreactive products were stained with 3,3′-diaminobenzidine and subsequently counterstained with hematoxylin. Finally, the sections were examined with a microscope (Q550CW; Leica, Manheim, Germany). For the evaluation of p57 and p-cofilin protein expression, the staining intensity was graded and scored as follows: 0, no staining; 1, weak staining; 2, moderate staining; and 3, strong staining. The extent of the staining was graded as 1 (≤25 %), 2 (26–50 %), 3 (51–75 %) and 4 (≥76 %) based on the percentage of positively stained cells [12]. The number of positive cells was assessed by counting 10 random fields at × 400 magnification. The final immunohistochemical staining score was obtained by multiplying the intensity and the extent of staining: A score of 0–2 was considered to be negative expression; 3–5, weak expression; 6–9, moderate expression; and 10–12, strong expression. A score of 6–12 was defined as positive staining, a score of 0–5 was defined as markedly reduced expression or a lack of expression.

### Plasmids

The p57 shRNA plasmids (pGPU6/GFP/Neo-shp57) were designed and synthesized by GenePharma Co. (Shanghai, China). The optimal p57 shRNAs had the following sequences: 5′-CACCGCTTTAAGAGTCATTTATATTCAAGAGATATAAATGACTCTTAAAGCTTTTTTG-3′ (sense) and 5′-GATCCAAAAAAGCTTTAAGAGTCAT TTATATCTCTTGAATATAAATGACTCTTAAAGC-3′ (antisense). The shRNA expression vector was pGPU6/GFP/Neo (GenePharma). The plasmid pGPU6/GFP/Neo-shNC, which encodes a hairpin siRNA with a sequence that is not found in human genome databases, was used as a negative control.

### Cell culture, construction of stable transfectants and treatments

The hepatoma cell lines HepG2, Hep3B, BEL7402, SMMC7721, and MHCC97H and the normal liver cell line L02 were obtained from Shanghai Cell Bank and cultured in DMEM (Invitrogen, Carlsbad, CA, USA) supplemented with 10 % FBS (Invitrogen), penicillin (100 IU/ml) and streptomycin (0.1 mg/ml) at 37 °C with 5 % CO_2_. BEL7402 cells and SMMC7721 cells were selected for our research. The cells were transfected with shp57 and shNC plasmids using Lipofectamine 2000 (Invitrogen) according to the manufacturer’s protocols. After 24 h, the cells were diluted and selected for 1 month using G418 (Invitrogen) at 600 μg/ml for BEL7402 cells and 800 μg/ml for SMMC7721 cells. Stable transfectants were confirmed by western blot and were maintained in medium containing G418 at 300 μg/ml for BEL7402 cells and 400 μg/ml for SMMC7721 cells. The stable p57 knockdown transfectants were named BEL7402-shp57 and SMMC7721-shp57. HCC cells transfected with shNC were named BEL7402-shNC and SMMC7721-shNC, which were used as controls.

### Reverse transcription PCR

Total mRNA was extracted using TRIzol reagent (Invitrogen), and reverse transcription was performed using an RT-PCR kit (Takara, Dalian, China). cDNA synthesis was conducted with the SYBR ExScript RT-PCR kit (Takara) according to the manufacturer’s instructions. The PCRs consisted of 5 min at 94 °C followed by 30 cycles of denaturation for 30 s at 94 °C, annealing for 30 s at 58 °C and primer extension for 30 s at 72 °C. The primer sequences for p57 were 5′-GCGGCGATCAAGAAGCTGT-3′ and 5′-ATCGCCCGACGACTTCTCA-3′. The primer sequences for glyceraldehyde 3-phosphate dehydrogenase (GAPDH) were 5′-ACCACAGTCCATGCCATCAC-3′ and 5′-TCCACCACCCTGTTGCTGTA-3′. Each measurement was performed in triplicate. GAPDH was applied as the internal housekeeping gene control.

### Western blot

Cells were lysed using cell lysis buffer as previously reported [13]. Nuclear and cytoplasmic extracts were prepared as previously reported [14]. Equivalent amounts of protein were separated by SDS-PAGE (8–12 %) and transferred onto polyvinylidene fluoride membranes (Millipore, Danvers, MA, USA). The membranes were blocked and subsequently incubated with the following primary antibodies: anti-p57 antibody (1:500), anti-phospho-cofilin (Ser3) antibody (1:300, 11139, Signalway Antibody, Pearland, TX, USA), anti-β-actin antibody (1:1000, sc-130301, Santa Cruz) and anti-lamin A antibody (1:600, 613501, Biolegend, San Diego, CA, USA). Blots were visualized with a secondary antibody conjugated to horseradish peroxidase (Santa Cruz) and an ECL detection system (Millipore). Western blotting was repeated three times for each protein.

### Co-immunoprecipitation and immunoblotting

Cells were harvested and lysed in 0.1 % NP-40 lysis buffer (50 mM Tris, pH 7.4, 250 mM NaCl, 5 mM ethylenediaminetetraacetic acid, 50 mM NaF, 1 mM Na_3_VO_4_, 0.1 % NP-40, 0.02 % NaN_3_, 1 mM phenylmethylsulfonyl fluoride). For immunoprecipitation, 2 μg of anti-p57 antibody was incubated with 50 μl of Dyna-beads/protein G (Invitrogen) for 10 min at room temperature and subsequently incubated with 500-μl cell lysate samples for 10 min at room temperature with gentle rotation to form Dynabead-Ab-Ag complexes. These complexes were then washed extensively with phosphate-buffered saline, resolved using 10 % sodium dodecyl sulfate–polyacrylamide gel electrophoresis and immunoblotted with anti-LIMK1 antibody.

### Confocal immunofluorescence analysis

Cells (1 × 10^5^ per well) were fixed with 4 % paraformaldehyde and blocked with 5 % bovine serum and 0.3 % Triton X-100. The cells were then incubated with anti-p57 antibody (1:100) and anti-phospho-cofilin (Ser3) antibody (1:50) at 4 °C overnight followed by staining with TRITC-conjugated anti-rabbit immunoglobulin (1:50, Santa Cruz) and 1 μg/ml DAPI (Roche). Immunofluorescence was visualized with a confocal laser scanning microscope (TCS SP5, Leica, Germany).

### Statistical analysis

Descriptive data were assessed using Pearson’s chi-square test (two-sided). Quantitative data were assessed by Student’s *t*-test. *P* < 0.05 was considered to be statistically significant. All statistical analyses were performed using SPSS 17.0 software for Windows (Chicago, IL).

## Results

### Relationship between the subcellular localization of p57 protein and clinicopathologic factors of HCC

**Fig. 1:**
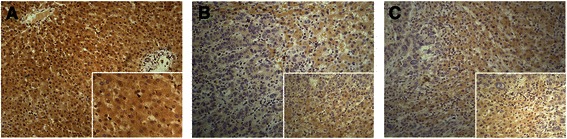
Immunohistochemical staining of p57 and p-cofilin protein in cancerous tissues and adjacent non-cancerous tissues of hepatocellular carcinoma patients (×200). **a** Positive expression of p57 in both the nucleus and the cytoplasm of adjacent non-cancerous tissues. **b** In contrast with adjacent non-cancerous tissues, positive p57 expression in the cytoplasm is decreased in the specimen with extrahepatic metastasis. **c** Positive p-cofilin expression in the cytoplasm is decreased compared to adjacent non-cancerous tissues in the specimen with extrahepatic metastasis. Each inset shows images taken at × 400 magnification

To determine the subcellular localization of p57 protein, immunohistochemistry was performed on 45 human HCC tissues and adjacent non-cancerous tissues. We noticed that p57 was expressed in both the nucleus and cytoplasm in the HCC tissues and the adjacent non-cancerous liver tissues (Fig. 1a, b). In the HCC tissues, positive expression of p57 was detected in the nucleus in 14 samples and in the cytoplasm in 17 samples (Additional file 1: Figure S1). To determine the association between subcellular localization and p57 function, we further analyzed the relationship of the nuclear and cytoplasmic p57 with clinicopathological factors. The results showed that nuclear expression of p57 was reduced in specimens from patients with smaller tumors (*P* < 0.05) and that cytoplasmic expression of p57 was reduced in specimens from patients with capsular invasion and metastasis (*P* < 0.05) (Table 1). These data indicated that the cytoplasmic localization of p57 may have a biological function related to HCC invasion.

### Association of cytoplasmic expression of p57 with p-cofilin in HCC tissues

It is well known that LIMK1 phosphorylates cofilin at Ser-3 and blocks the ability of cofilin to depolymerize actin filaments during cell migration [15]. Our previous data suggested that p57 downregulation accelerated the invasion of HCC cells by controlling the activity of LIMK1 and subsequently reducing the phosphorylation of cofilin [11]. To further elucidate whether p57 regulates HCC cell invasion in the cytoplasm, immunohistochemistry of p-cofilin was performed in human HCC tissues. The results indicated that p-cofilin was localized only in the cytoplasm (Fig. 1c). Moreover, we observed that of the samples with increased cytoplasmic p57 expression, 14 samples were positive for p-cofilin expression, and of the samples with decreased cytoplasmic p57 expression, 15 samples were negative for p-cofilin expression (*P* < 0.05). We further analyzed the association of cytoplasmic co-expression of p57 and p-cofilin with clinicopathological factors. The results showed that cytoplasmic co-expression of p57 and p-cofilin was reduced in specimens from patients with tumors at later stages (III + IV) and patients with capsular invasion and metastasis (*P* < 0.05) (Table 2). These data suggest that p57 and p-cofilin might function in the cytoplasm to regulate HCC invasion.

**Table 2:** Association of cytoplasmic co-expression of p57 and p-cofilin with clinicopathologic factors in cancerous tissues of hepatocellular carcinoma patients

Variable	n	Cytoplasmic co-expression of p57 and p-cofilin		*P*
		Positive	Non-positive	
Tumor size				0.512
<50 mm	18	7	11	
≥50 mm	27	7	20	
Histological grade				0.428
I + II	36	10	26	
III	9	4	5	
TNM stage				0.004
I + II	12	8	4	
III + IV	33	6	27	
Capsular invasion and extrahepatic metastasis				0.009
Positive	24	3	21	
Negative	21	11	10	
AFP				0.520
<400 μg · L^−1^	21	8	13	
≥400 μg · L^−1^	24	6	18	

*n* Number of patients, *AFP* alpha-fetoprotein, *TNM* tumor-node-metastasis

### p57 regulates the level of p-cofilin in the cytoplasm of HCC cells

**Fig. 2:**
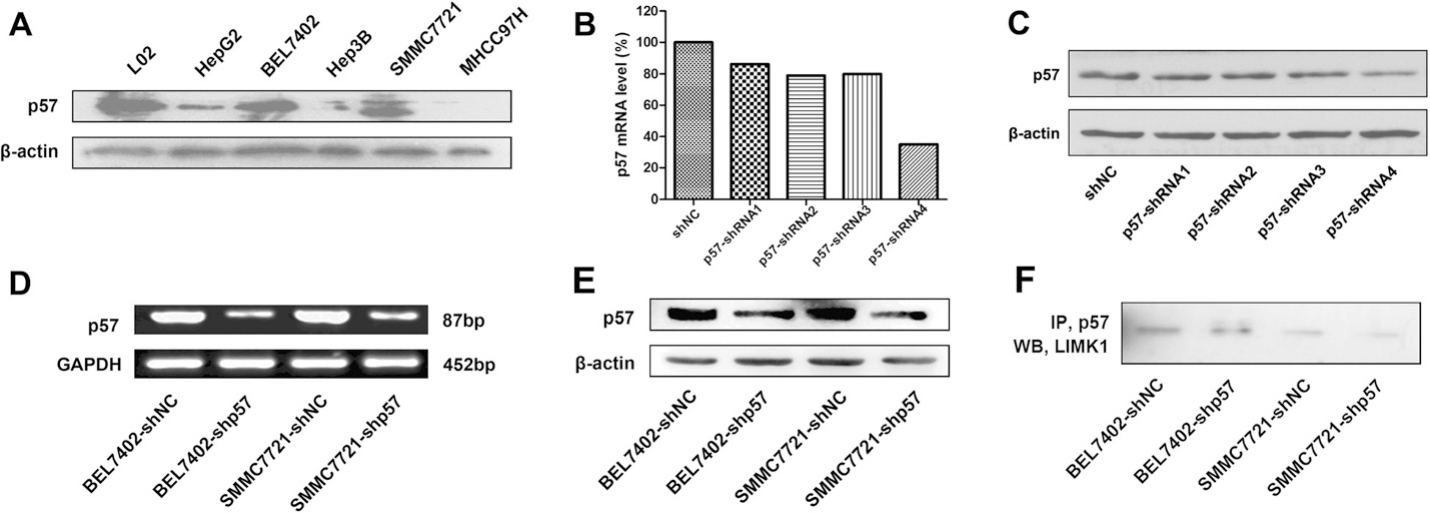
p57 knockdown in hepatoma cell lines and p57 interaction with LIM domain kinase 1. **a** Western blot analysis of p57 expression in hepatoma cell lines and a normal liver cell line. **b** Reverse transcription polymerase chain reaction amplification of p57 mRNA in BEL7402 cells transfected with four p57 shRNAs. **c** Western blot analysis of p57 expression in BEL7402 cells transfected with four p57 shRNAs. **d** Reverse transcription polymerase chain reaction amplification of p57 mRNA in BEL7402-shNC, BEL7402-shp57, SMMC7721-shNC and SMMC7721-shp57 cells. **e** Western blot detection of p57 protein in BEL7402-shNC, BEL7402-shp57, SMMC7721-shNC and SMMC7721-shp57 cells. **f** Co-immunoprecipitation was used for the detection of the association between p57 and LIM domain kinase 1 in hepatoma cell lysates following different treatments. β-actin was used as an internal control. Glyceraldehyde 3-phosphate dehydrogenase was applied as the internal housekeeping gene control. The expression of each protein was detected by western blot in at least three separate experiments, and representative images are shown. LIMK1, LIM domain kinase 1; GAPDH, glyceraldehyde 3-phosphate dehydrogenase; IP, co-immunoprecipitation; WB, western blot

We next investigated the role of p-cofilin protein in p57-knockdown hepatoma cell lines. We selected the BEL7402 and SMMC7721 cell lines for the follow-up experiments because they exhibit moderate p57 expression, in contrast with the normal liver cell line LO2 (Fig 2a). Out of four p57 shRNAs, we selected p57-shRNA4 because it induced optimal downregulation of p57 mRNA and protein (Fig 2b, c). Stable transfectants with downregulated p57 expression were constructed, and we observed that the level of p57 mRNA and protein was decreased in BEL7402-shp57 and SMMC7721-shp57 compared to BEL7402-shNC and SMMC7721-shNC (Fig 2d, e). Our previous experiments showed that p57 did not affect the level of LIMK1 but could affect the level of p-cofilin, which is the unique substrate of LIMK1 and reflects its activity [11]. However, the mRNA level of cofilin has no change in BEL7402-shp57 and SMMC7721-shp57 compared to BEL7402-shNC and SMMC7721-shNC cells (Additional file 2: Figure S2). We questioned whether p57 could directly bind LIMK1 to affect its activity. By co-immunoprecipitation, we further proved that p57 downregulation inhibited the assembly of p57 and LIMK1, indicating that p57 could combine with LIMK1 and regulate its kinase activity (Fig. 2f).

**Fig. 3:**
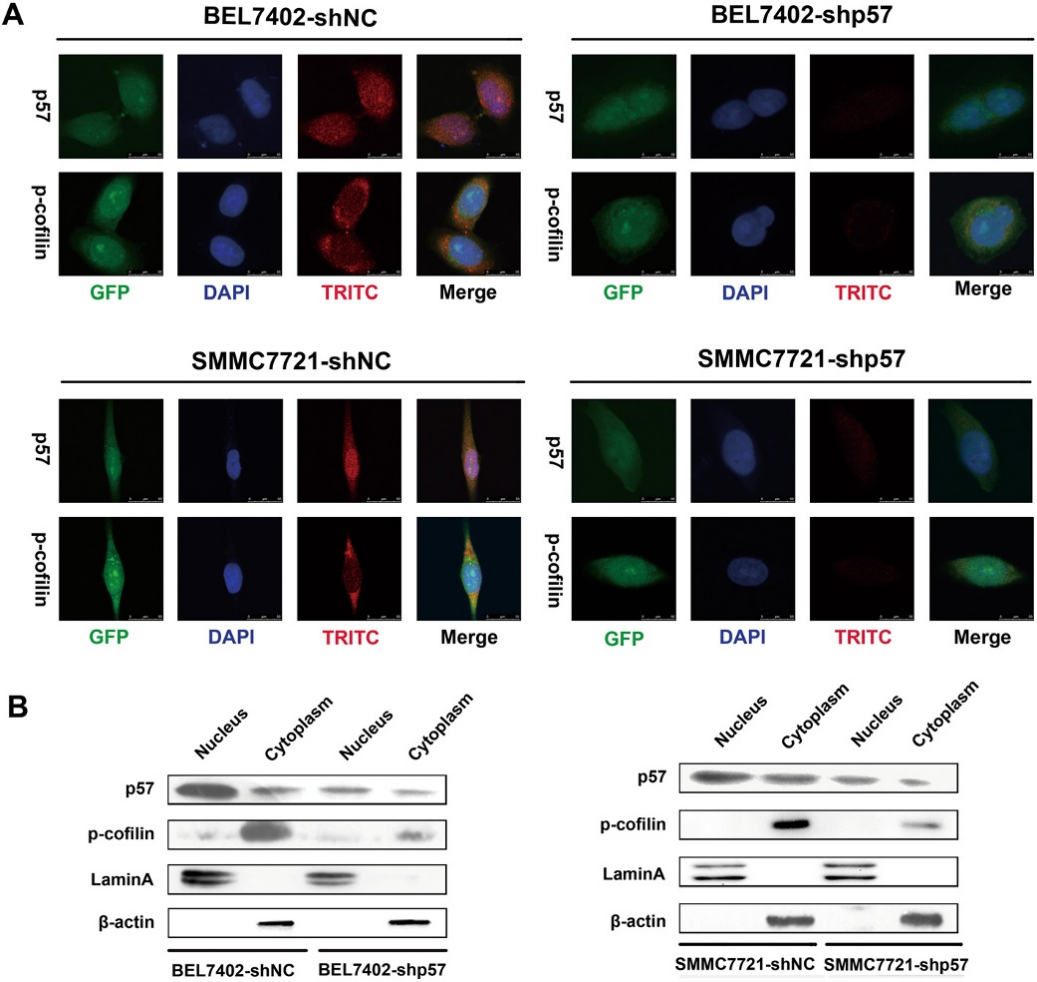
p57 regulates the expression of p-cofilin in the cytoplasm. **a** Confocal immunofluorescence microscopy analysis of BEL7402-shNC, BEL7402-shp57, SMMC7721-shNC and SMMC7721-shp57 cells. Green fluorescence represents the transfected plasmid, red fluorescence shows the expression of the target protein, and blue fluorescence shows the nucleus. **b** Western blot analysis of nuclear and cytoplasmic extracts of p-cofilin in BEL7402-shNC, BEL7402-shp57, SMMC7721-shNC and SMMC7721-shp57 cells. β-actin was used as an internal control for cytoplasmic proteins. Lamin A was used as an internal control for nuclear proteins. GFP, Green fluorescent protein; TRITC, tetramethylrhodamine; DAPI, 4′,6-diamidino-2-phenylindole

To further verify whether p57 regulates the activity of LIMK1 in the cytoplasm, confocal immunofluorescence microscopy and western blot analysis of nuclear and cytoplasmic extracts were performed. In BEL7402-shNC and SMMC7721-shNC cells, we observed that p57 was expressed in both the nucleus and the cytoplasm, whereas p-cofilin was only expressed in the cytoplasm. p57 downregulation decreased the level of p-cofilin in the cytoplasm in BEL7402-shp57 and SMMC7721-shp57 cells (Fig. 3a, b). These findings demonstrated that p57 might affect the level of p-cofilin by regulating the kinase activity of LIMK1 in the cytoplasm.

## Discussion

Increasing evidence has indicated that the Cip/Kip family of proteins has a role in cell migration and tumor metastasis [7]. Some clinical studies have shown that decreased expression of p57 is related to increased invasion and metastasis in cancers, categorizing this protein as a suppressor of tumor metastasis [16, 17]. Inducible expression of p57 in glioma cell lines deficient in this CKI reduces their motility and invasion [18]. In addition, p57 is involved in angiogenesis, which is an important event in cancer metastasis, via the regulation of vascular endothelial growth factor (VEGF) mRNA and protein levels [19]. p21 and p27, which are other Cip/Kip family members, were shown to regulate cell motility and invasion in the cytoplasm. However, the role of cytoplasmic p57 and its relationship with biological functions are unclear. Our study showed that p57 was localized to both the nucleus and the cytoplasm of normal liver cells and HCC cells. A correlation analysis indicated that nuclear p57 was reduced in specimens from patients with smaller tumors, and cytoplasmic p57 was reduced in specimens from patients with capsular invasion and metastasis. These results suggest that nuclear localization of p57 might regulate cellular proliferation and that cytoplasmic localization of p57 might regulate cell motility. However, the mechanism of action of p57 is different from that of p21 and p27. Cytoplasmic p21 was found to inhibit the activity of ROCK1, reduce the formation of actin stress fibers, and promote the invasion and metastasis of tumor cells [20]. In HCC, melanoma and breast cancer cells, cytoplasmic expression of p27 was found to promote migration and metastasis by inhibiting RhoA activity [21]. These findings indicate that cytoplasmic p21 and p27 might play a role as oncogenes in some human cancers. However, our results showed that cytoplasmic p57 inhibits the invasion and metastasis of HCC.

Cofilins are actin-binding proteins that play an essential role in regulating actin filament dynamics and reorganization by stimulating the severing and depolymerization of actin filaments in cell motility and cancer metastasis [22]. It is well known that cofilin is inactivated by phosphorylation by LIMK1, and the level of p-cofilin can reflect the activity of LIMK1. The interaction between p57 and LIMK1/p-cofilin has been reported in human cells [23, 24]. In HCC tissues, we found that cytoplasmic p57 was positively correlated with cytoplasmic p-cofilin. More importantly, co-expression of p57 and p-cofilin was reduced in specimens from patients with tumors at later stages (III + IV) and patients with capsular invasion and metastasis. Moreover, we found that p57 downregulation decreased the assembly of p57 and LIMK1. The results of confocal immunofluorescence microscopy and western blot analysis of nuclear and cytoplasmic extracts further supported the hypothesis that p57 could regulate the level of p-cofilin in the cytoplasm. These data suggested that p57 might regulate the kinase activity of LIMK1 and subsequently reduce the level of p-cofilin in the cytoplasm to regulate HCC invasion.

Thus far, the mechanism regulating the subcellular localization of p57 has remained undefined, although it has been reported that phosphorylation and ubiquitination are two main mechanisms involved in this process. In breast cancer, Akt was found to interact with p57 and cause cytoplasmic localization of p57 by phosphorylating its Ser282 or Thr310 residue. Akt activity resulted in destabilization of p57 by accelerating the turnover rate of p57 and enhancing p57 ubiquitination. Importantly, the negative impact of HER2/Akt on p57 stability contributed to HER2-mediated cell proliferation, transformational activity and tumorigenicity [25]. These findings suggested that the AKT pathway might regulate p57 subcellular localization and subsequently affect its biological function. Importantly, phosphorylation is often coordinated with ubiquitination in the process of p57 degradation. In osteoblast cells, FBL12 was found to form an SCF (FBL12) complex and directly ubiquitinate p57, causing p57 degradation in a phosphorylation-dependent manner [26]. Another E3 ligase, Skp2, could also mediate the ubiquitination of p57 by collaborating with CSN6 in the cytoplasm to regulate tumor progression [27]. These studies indicated that p57 might be exported from the nucleus due to phosphorylation and degraded in the cytoplasm due to ubiquitination during tumor growth and invasion.

## Conclusions

Our data indicated that reduced cytoplasmic p57 expression is associated with HCC invasion. Furthermore, p57 inhibits HCC invasion by regulating the level of p-cofilin in the cytoplasm via interaction with LIMK1, suggesting that cytoplasmic p57 may be a key regulator of HCC invasion. However, some questions remain concerning the mechanisms by which the subcellular localization of p57 regulates HCC progression. In the future, we will further investigate the mechanism of nuclear-cytoplasmic shuttling of p57. Additionally, we will explore components of this mechanism as promising targets for HCC prevention and therapy.

## Additional files

Additional file 1: Figure S1. Immunohistochemical staining of nuclear p57 protein in adjacent non-cancerous tissues (left) and cancerous tissues (right) of hepatocellular carcinoma patients (×200). Additional file 2: Figure S2. p57 downregulation can not affects the mRNA level of p-cofilin in HCC cell lines. Reverse transcription PCR analysis of BEL7402-shNC, BEL7402-shp57, SMMC7721-shNC and SMMC7721-shp57 cells.

## Abbreviations

CKI: Cyclin-dependent kinase inhibitor
RhoA: Ras homolog gene family, member A
ROCK1: Rho-associated, coiled-coil-containing protein kinase 1
LIMK1: LIM domain kinase 1
HCC: Hepatocellular carcinoma
VEGF: Vascular endothelial growth factor
UICC: International union against cancer
HE: Hematoxylin/eosin
GAPDH: Glyceraldehyde 3-phosphate dehydrogenase
AFP: Alpha-fetoprotein
TNM: Tumor-node-metastasis
