# Supplementary figures and images for: The role of cytoplasmic p57 in invasion of hepatocellular carcinoma
Source: BMC Gastroenterol. 2015 Aug 15;15:104. doi: 10.1186/s12876-015-0319-x (PMC4542127; doi:10.1186/s12876-015-0319-x)

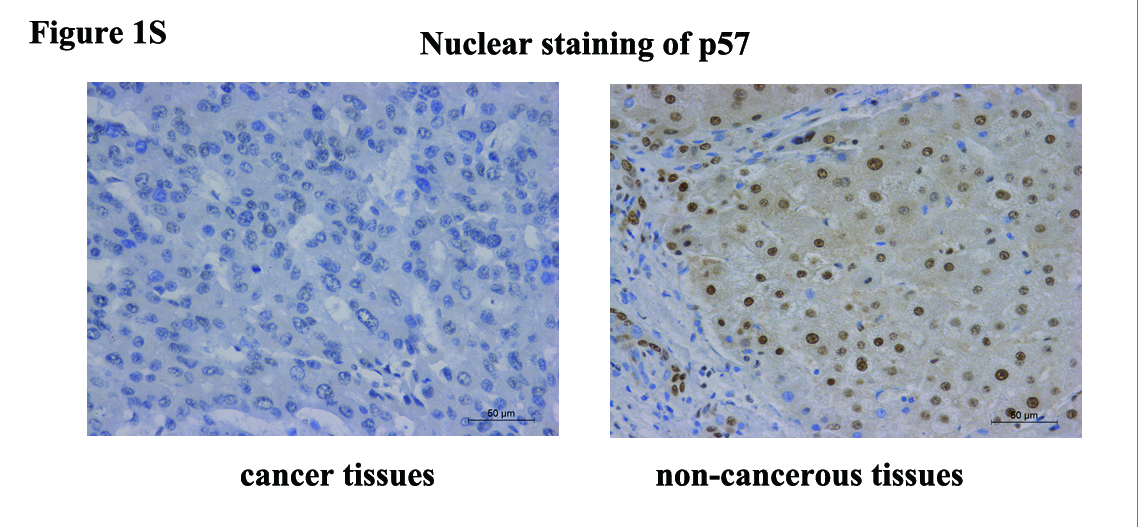

Supplement: Additional file 1: Figure S1. — Immunohistochemical staining of nuclear p57 protein in adjacent non-cancerous tissues (left) and cancerous tissues (right) of hepatocellular carcinoma patients (×200). [file 12876_2015_319_MOESM1_ESM.tiff]

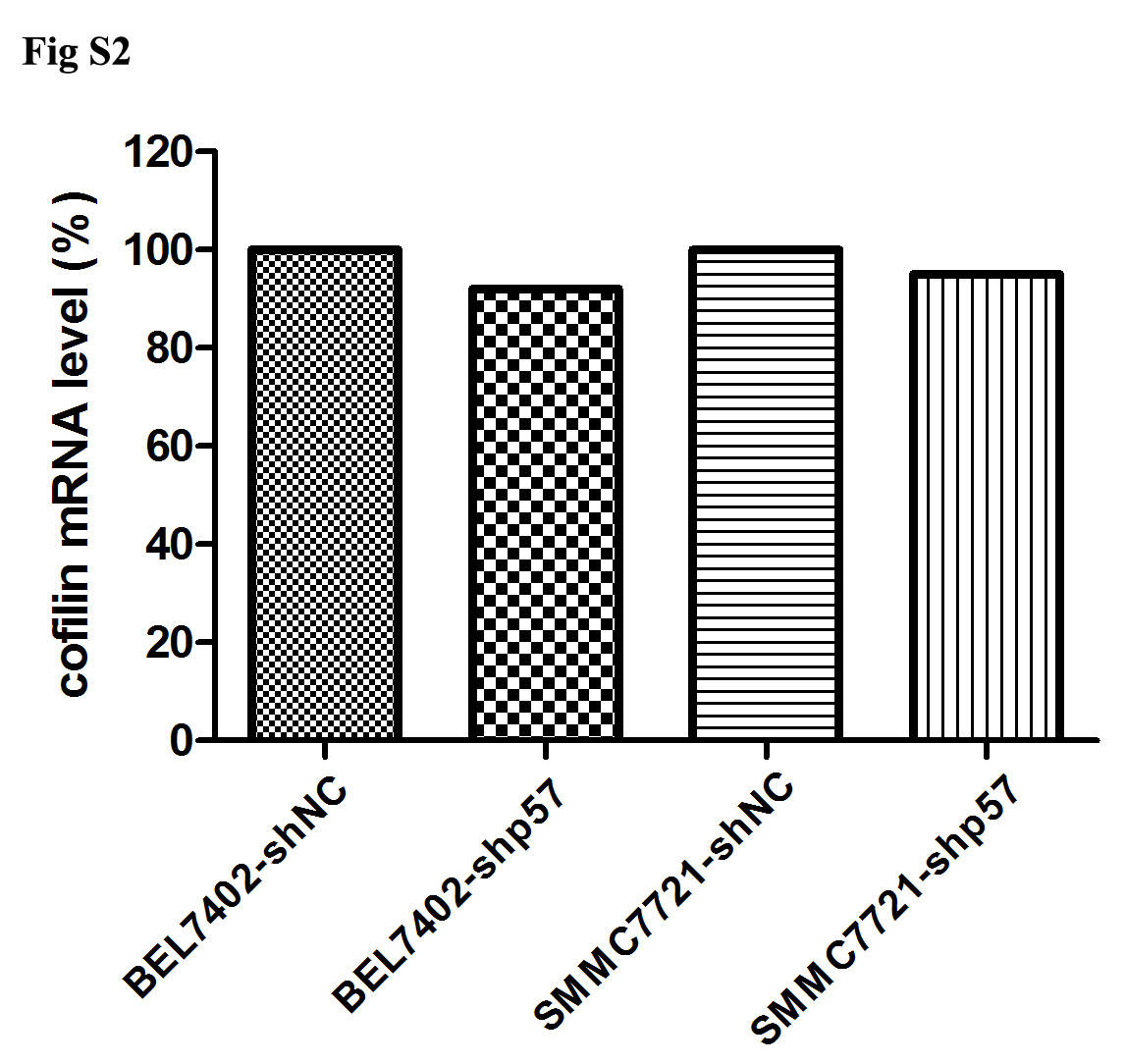

Supplement: Additional file 2: Figure S2. — p57 downregulation can not affects the mRNA level of p-cofilin in HCC cell lines. Reverse transcription PCR analysis of BEL7402-shNC, BEL7402-shp57, SMMC7721-shNC and SMMC7721-shp57 cells. [file 12876_2015_319_MOESM2_ESM.jpeg]
